# Cross-Lagged Relationship Between Adiposity and HOMA and Mediating Role of Adiposity Between Lifestyle Factors and HOMA Among in Mexican Health Workers

**DOI:** 10.3390/nu17152497

**Published:** 2025-07-30

**Authors:** Joacim Meneses-León, Amado D. Quezada-Sánchez, Mario Rojas-Russel, Diana I. Aparicio-Bautista, Rafael Velázquez-Cruz, Carlos A. Aguilar-Salinas, Jorge Salmerón, Berenice Rivera-Paredez

**Affiliations:** 1 Research Center in Policies, Population and Health, School of Medicine, National Autonomous University of Mexico, Mexico City 04510, Mexico; ml.joacim@gmail.com (J.M.-L.);; 2 Center for Evaluation and Surveys Research, National Institute of Public Health, Cuernavaca 62100, Mexico; 3 Facultad de Estudios Superiores Zaragoza, National Autonomous University of Mexico, Mexico City 09230, Mexico; mario.rojas@zaragoza.unam.mx; 4 Genomics of Bone Metabolism Laboratory, National Institute of Genomic Medicine (INMEGEN), Mexico City 14610, Mexico; 5 Research Unit for the Study of Metabolic Diseases, Instituto Nacional de Ciencias Médicas y Nutrición Salvador Zubirán, México City 14080, Mexico; 6 Tecnológico de Monterrey, Escuela de Medicina y Ciencias de la Salud, Mexico City 64710, Mexico

**Keywords:** lifestyle factors, IR, adiposity, SEM, cross-lagged panel model, Mexican adults

## Abstract

**Background/Objectives**: Unhealthy lifestyles are closely linked to insulin resistance (IR) and adiposity. However, the mediating role of adiposity in the relationship between lifestyle factors and IR is not yet fully understood. Mediation analysis may help clarify the role of adiposity in the relationship between lifestyle factors and IR. Therefore, we aimed to explore the bidirectional relationship between adiposity and IR, and to evaluate the relationship between lifestyle factors and adiposity-mediated IR in Mexican adults. **Methods**: A longitudinal analysis was conducted using data from the Health Workers Cohort Study, with measurements taken every six years from 2004 to 2018. This study included 1134 participants aged from 18 to 70 years. Lifestyle factors were assessed using a self-administered questionnaire. IR was assessed using the Homeostasis Model Assessment (HOMA). Adiposity was measured through body mass index (BMI), waist circumference (WC), and body fat proportion (BFP), and BMI was used as the marker indicator to set the metric of adiposity. We fitted structural equation models with a cross-lagged specification to examine the relationships between adiposity and ln(HOMA). In our analysis, we considered baseline adiposity and ln(HOMA) as mediators of the relation between lifestyle factors and future adiposity and ln(HOMA). Models were stratified by sex and adjusted by baseline age. **Results**: Results from the cross-lagged panel model showed that, for both men and women, adiposity predicted subsequent increases in HOMA (+5.3% IC95%: 1.8%, 9.0% in men; +6.0% IC95%: 4.2%, 7.8% in women). In men, baseline adiposity acted as a mediator between lifestyle variables (physical activity, tobacco consumption, and sleep duration) and HOMA. **Conclusions**: Our results suggest that understanding both the relationship between adiposity and HOMA and the mediating effects of adiposity is crucial for developing effective interventions to reduce IR in the Mexican population.

## 1. Introduction

Insulin resistance (IR) is characterized by a reduced capacity of insulin to transport and utilize glucose in muscle and adipose tissue, as well as impaired suppression of hepatic gluconeogenesis [1]. This metabolic dysfunction can lead to various metabolic disorders, including hypertension, dyslipidemia, hyperuricemia, cardiovascular disease, type 2 diabetes (T2D), and metabolic syndrome [1,2,3,4,5]. In recent years, the prevalence of IR has been increasing. According to the National Health and Nutrition Examination Survey (NHANES), 40% of American adults are insulin-resistant [6], while, in Mexico, 21.2% of a population of health workers adults were estimated to have IR [5]. Obesity is considered one of the most critical risk factors for IR, and Mexico has one of the highest adult obesity prevalence rates globally [7].

Factors associated with IR include genetic predisposition, aging, and lifestyle-related behaviors, such as physical inactivity, unhealthy dietary patterns, tobacco and alcohol consumption, insufficient sleep, and psychosocial factors like stress and depression [8,9,10,11,12]. The mechanism by which adiposity influences IR risk involves obesity-induced adipocyte hypertrophy, which impairs preadipocyte differentiation. This process leads to increased triglyceride accumulation in adipose tissue, promoting macrophage infiltration and the activation of innate immune cells. These events result in hyperinsulinemia, which, in turn, inhibits lipolysis and activates lipoprotein lipase [2,13,14]. Consequently, this results in increased hyperinsulinemia, hypertriglyceridemia, heightened IR in muscle and adipose tissue, and impaired adipogenesis [1,2,13,14].

Several studies have described the individual associations between various risk factors for IR [8,9,10,11,12]. More recently, some research has evaluated the association between lifestyle factors and IR, mediated through adiposity [15,16,17]. However, these studies have not modeled a reciprocal relationship between adiposity and IR in which mediation could occur through either factor. Mediation analysis using structural equation modeling (SEM) is particularly useful for exploring these complex relationships, including multiple pathways between lifestyle factors and IR (or adiposity) through adiposity (or IR) [18]. This approach is crucial because it clarifies the paths through which lifestyle choices affect IR, potentially identifying targets for intervention.

Cross-sectional studies have found that individuals with lower adherence to the Mediterranean diet and those engaging in low-intensity physical activity are at a higher risk of IR mediated by waist circumference (WC) [15,16]. Additionally, a longitudinal study reported a positive association between the Alternate Healthy Eating Index (AHEI) and WC-mediated IR, suggesting that reducing abdominal adiposity through lifestyle changes may lower the risk of IR [17]. Despite these findings, robust longitudinal studies examining the association between different lifestyle factors and the risk of IR mediated by adiposity are still lacking and are needed to better understand causal pathways and inform effective prevention strategies [18]. Given the potential for lifestyle interventions to mitigate IR, understanding these relationships is crucial for effective public health strategies. Furthermore, some studies have reported that abdominal obesity has greater metabolic effects than general obesity, as measured by body mass index (BMI) [19], highlighting the importance of considering multiple measures of adiposity simultaneously. This simultaneous consideration would reduce measurement error. Additionally, it is important to address multiple lifestyle factors, as each may capture specific aspects with heterogeneous associations with adiposity and IR.

According to the Mexican National Health and Nutrition Survey (ENSANUT), the Mexican population is undergoing an epidemiological transition that is contributing to the national burden of disease [20,21]. Therefore, it is crucial to provide epidemiological evidence regarding the association between lifestyle factors and IR, a health issue that remains understudied in our country. While obesity (elevated adiposity) is a well-established risk factor for the development of IR, there is also evidence suggesting that IR may promote abdominal fat accumulation, creating a vicious cycle that perpetuates both conditions through cell-autonomous mechanisms or by inducing inflammation and the subsequent production of inflammatory cytokines by macrophages, which impair insulin action [22,23]. Understanding the reciprocal effects between these two factors is essential, as it can provide insight into how changes in one factor may impact the other over time. Additionally, analyzing adiposity as a latent variable in a SEM approach, measured by multiple indicators, such as BMI, WC, and body fat percentage (BFP), has the advantage of taking into account measurement error in the estimation of model parameters. In contrast, classical linear regression assumes no measurement error in explanatory variables. Therefore, this study aims to evaluate the bidirectional relationship between adiposity and IR, and to examine how adiposity mediates the relationship between lifestyle factors and IR in Mexican adults.

## 2. Materials and Methods

### 2.1. Study Population

We used data from the Health Workers Cohort Study (HWCS), a dynamic prospective cohort study involving workers and their families [24]. Participants were recruited from the Mexican Social Security Institute (IMSS) in Cuernavaca Morelos, Mexico. The HWCS aims to examine the associations between genetic factors, lifestyle behaviors, and various health outcomes.

This analysis included data from 2048 individuals aged 18 years and older. Between 2004 and 2006, 1849 men and women aged from 18 to 70 years participated in the initial evaluation. An additional 199 participants in the same range were enrolled between 2010 and 2012. Follow-up assessments were conducted approximately every six years using self-administered questionnaires and standardized procedures. Participants were excluded for the analysis for following reasons: pregnancy (*n* = 34), missing data on the homeostasis model assessment of IR (HOMA-IR) (*n* = 425), incomplete dietary data (defined as answering less than 75% of the items or not completing any section of the food frequency questionnaire) (*n* = 75), extreme caloric intake values (<600 or >7000 Kcal/day, defined as >3.86 SD using the standard deviation method [25]) (*n* = 17), and missing data on physical activity (*n* = 2), smoking status (*n* = 3), sleep duration (*n* = 31), and depressive symptoms (*n* = 81). Additionally, participants with diagnosed health conditions at baseline were excluded, including those with T2D (previously diagnosed or newly identified cases and current use of diabetes medications) (*n* = 106), kidney disease (*n* = 38), cancer (*n* = 42), or cardiovascular disease (*n* = 60). After applying these exclusion criteria, a total of 1134 participants with at least two measurements were included in the final analysis.

### 2.2. Demographic Characteristics and Lifestyle Factors

Demographic data were collected using a self-administered questionnaire. Smoking status was categorized according to World Health Organization criteria as non-smoker, ex-smoker, and current smoker [26].

### 2.3. Physical Activity

Physical activity (PA) was assessed using a self-administered questionnaire validated in Spanish for similar populations [27]. Participants reported the time spent per week on leisure-time activities, such as walking, running, and cycling, during a typical week in the previous year. PA was expressed in minutes/day [27,28]. Based on this, participants were categorized as either inactive (<30 min/d) or active (≥30 min/d).

### 2.4. Sleep Time

Sleep time (ST) was assessed using a self-administered questionnaire. Participants reported their average daily ST and nap duration. ST was expressed in hours/day and nap in minutes/day [29]. The nap variable was categorized as yes or no.

### 2.5. Depressive Symptoms

Depressive symptoms (DS) were assessed using the Center for Epidemiological Studies Depression Scale (CES-D), which includes 20 items evaluating the frequency of depressive symptoms experienced over the past week. The items cover affective, interpersonal, somatic, and behavioral domains [30]. Responses are scored on a Likert scale, ranging from 0 (rarely [less than 1 day]) to 3 (most or all of the time [5–7 days]), yielding a total score ranging from 0 to 60 points. A score below 16 indicates the absence of depressive symptoms, while a score of 16 or above suggests clinically significant DS [30].

### 2.6. Dietary Assessment

Diet was assessed using a semi-quantitative food frequency questionnaire (FFQ) validated for the Mexican population. The FFQ collected data on the frequency of consumption of 116 food items over the previous year [31]. Reported intake data were then converted into estimates of daily caloric (Kcal/day) and nutrient intake using the Nutritional Habits and Nutrient Consumption Assessment System (SNUT) [32].

### 2.7. Dietary Inflammatory Index

Dietary inflammatory index (DII) scores were calculated following the methodology proposed by Shivappa et al. [33]. Each participant’s DII score was derived from 30 of the 45 possible dietary parameters, including total energy, macronutrients (carbohydrates, protein, total fat, saturated fat, trans fat, monounsaturated and polyunsaturated fatty acids, omega 3 and 6), cholesterol, alcohol, fiber, caffeine, and micronutrients (iron, magnesium, niacin, riboflavin, selenium, thiamine, beta-carotene, zinc, folate, onion, and vitamins A, C, D, E, B6, and B12). A higher DII score indicates a pro-inflammatory diet, whereas a lower score reflects an anti-inflammatory diet [34]. To minimize the influence of total energy intake, nutrient intakes were energy-adjusted using the residual method [35].

### 2.8. Insulin Resistance

Venous blood samples were collected after an eight-hour fast. Serum glucose concentrations were determined on each occasion using the oxidized glucose method, while insulin levels were determined using a direct solid-phase radioimmunoassay. IR was estimated using the Homeostasis Model Assessment of Insulin Resistance (HOMA-IR), calculated as: HOMA-IR = [insulin (mIU/L) × glucose (mmol/L)/22.5] [36]. For the classification of subjects, we considered a HOMA > 3.2 [5].

### 2.9. Anthropometric Assessment

Body weight was measured using a calibrated electronic scale (TANITA; model BC-533, Tokyo, Japan) with participants wearing minimal clothing and without shoes. Height was measured with a conventional stadiometer (SECA), ensuring participants stood without shoes and maintained a neutral shoulder position [37]. BMI was calculated as weight (kg) divided by height squared (m^2^) [38]. BMI was classified as normal (<25 kg/m^2^), overweight (from ≥25 to <30 kg/m^2^), or obese (≥30 kg/m^2^) [38]. WC was measured at the highest point of the iliac crest at the end of normal expiration, to the nearest 0.1 cm, using a conventional tape measure (SECA). Abdominal obesity was defined as WC ≥ 102 cm for men and ≥88 cm for women [39]. Body fat proportion (BFP) was determined under fasting conditions using dual-energy X-ray absorptiometry (DXA) with a Lunar densitometer (model: DPX-GE 73735, serial: 638405U77) (Lunar Radiation Corporation, Madison, WI, USA; software version 1.35, fast scan mode) [40]. Excess BFP was defined as >35% in women and >25% in men [41]. All anthropometric and body composition measurements were performed by trained personnel following standardized methods [24].

### 2.10. Statistical Analysis

The enrollment visit served as the study baseline, encompassing either the 2004–2006 or 2010–2012 periods. For the descriptive analysis, measures of central tendency and dispersion were calculated for the baseline characteristics of the participants.

We specified a structural equation model (SEM) that included a measurement component for adiposity at each time point and a cross-lagged structure for adiposity and ln(HOMA). Adiposity was measured using continuous-type indicators (BMI, WC, and BFP) with BMI used as the marker indicator to set its scale; the constant was fixed to zero in the BMI equations. Given the longitudinal nature of this study, we imposed loading invariance across time and added correlation parameters between the errors of the same adiposity indicator across time. The cross-lagged structure for adiposity and ln(HOMA) implies that each of these outcomes at follow-up is explained by its baseline value (both baseline adiposity and ln(HOMA) in each follow-up equation). This specification allowed us to represent potential reciprocal effects between these two variables. We also set baseline adiposity and ln(HOMA) as mediators between baseline lifestyle characteristics and future adiposity and ln(HOMA). The natural log transform of the HOMA index served for two purposes, it made the HOMA distribution much less asymmetric and allowed us to interpret model parameters as geometric mean (GM) ratios when exponentiated [42]. From the GM ratios, it is also possible to express the relationships in terms of percentage changes in the outcome variable related to a unit increase in the explanatory variable through the equation $\%∆\;=100\times \left(e^{\beta }-1\right)$. On the other hand, when an explanatory variable is transformed with the natural log and the outcome is not transformed (level-log specification), its regression slope parameter can be used to approximate a change in the outcome associated with an increase of 1% in the explanatory variable when the parameter is divided by 100 [43,44]. We applied these interpretation properties to some of our results.

To account for omitted variables that may simultaneously explain adiposity and ln(HOMA), we specified correlation parameters between the errors of the adiposity and ln(HOMA) equations at each study time point. Exogenous model variables in our model were baseline lifestyle characteristics (physical activity, smoking status, sleep time, nap, and dietary inflammatory index), baseline depressive symptoms, and baseline age.

We fitted the SEM model for each sex separately in Mplus version 8 (Muthén & Muthén, Los Angeles, CA, USA) with the maximum likelihood method of estimation, which is implemented in conjunction with full information maximum likelihood. This procedure uses all available information of the measured outcomes to handle missing data and produces unbiased estimates when the missing mechanism is missing at random [45]. A total of 320 men and 814 women had complete information on all covariates, this sample had missing data only for WC (1.3% baseline and 3.1% follow-up for men; 1.2% baseline and 4.2% follow-up for women) and BFP (3.8% baseline and 10% follow-up for men; 3.9% baseline and 5.6% follow-up for women).

We calculated unstandardized specific indirect, total indirect and total effects, along with their standard errors. The full model equation parameters are presented unstandardized and with the STDY standardization; in the latter, only the endogenous variables and latent variables are standardized [46].

All other analyses and data preparation were conducted in Stata MP version 18.0 (Stata Corp. LP: College Station, TX, USA). The level of statistical significance was set at α = 0.05.

#### Goodness of Fit

We used the Comparative Fit Index (CFI), Tucker-Lewis Index (TLI), Root Mean Square Error of Approximation (RMSEA) with its 90% confidence interval (90%CI), and Standardized Root Mean Square Residual (SRMR) to assess model fit. We considered a very good fit when CFI/TLI was 0.95–0.99, an acceptable fit when 0.05 < RMSEA ≤ 0.08, and a good or close fit when RMSEA ≤ 0.05 [47]. An SRMR < 0.08 was considered a good fit [48]. For the adiposity measurement part of the model, we obtained the coefficient of determination (R^2^) for each adiposity indicator equation, which is an estimate of item reliability.

## 3. Results

### 3.1. Descriptive Characteristics of the Study Population

At baseline, the mean age was 42.9 (SD = 11.6); the majority were women (72.0%). The median HOMA was 1.6 [IQR: 0.7, 3.1] with a GM of 1.42 and 23.5% of participants classified as IR. The prevalence of overweight/obesity was 55.8%, while abdominal obesity and excess BFP were present in 36.3% and 81.6% of participants, respectively.

Regarding lifestyle factors, the mean of PA was 24.9 min/day (SD = 31.7), and the prevalence of current smokers was almost 19.0%. The medians of ST and napping were 7.3 h/day [IQR: 6.6, 8.0] and 7.5 min/day [IQR: 0.0, 32.1], respectively. Clinically significant depressive symptoms were present in 27.2% of individuals, and the median DII score was −0.78 [IQR: −1.50, 0.31] (Table 1).

### 3.2. Adiposity Measurement System and Bidirectional Relationships Between Adiposity and IR

**Figure 1:**
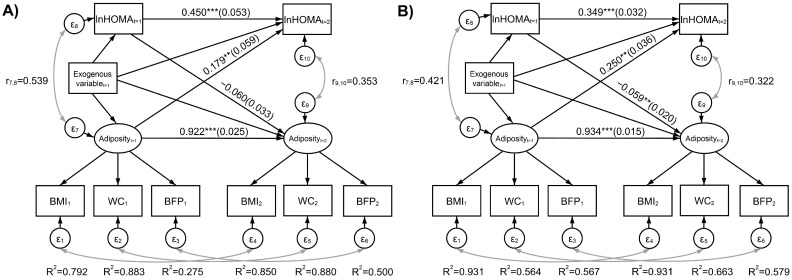
Simplified representation of the fitted structural equations models with a cross-lagged specification for adiposity and ln(HOMA), and initial adiposity and ln(HOMA) as mediators between lifestyle factors and future adiposity and ln(HOMA). Adiposity was measured from continuous-type indicators (BMI, WC, and BFP). Estimates are standardized coefficients with standard errors in parentheses. Exogenous variables: age, physical activity (≥30 min/day), smoking status (never, former smoker, and current smoker), sleep time (hours/day), nap (yes), DII (continuous), and depressive symptoms (≥30 points). Results on the left represent the relationship for (**A**) men, and on the right for (**B**) women. BMI: body mass index (kg/m^2^); WC: waist circumference (cm); BFP: total body fat mass proportion; R^2^: coefficient determination for the adiposity indicator equations, which is an estimate of reliability; ** *p* < 0.01; *** *p* < 0.001.

A simplified representation of the fitted SEM model, including standardized path coefficients, is presented in Figure 1. The reliability of adiposity indicators, as estimated by the R^2^ statistic, was highest for WC in men and for BMI in women, suggesting sex-related differences in adiposity expression. This pattern was similar across time points; however, baseline BFP reliability was comparatively low in men. Regarding the cross-lagged structure, baseline adiposity predicted a higher HOMA at follow-up for both men (0.179, 95% CI: 0.064, 0.295) and women (0.250, 95% CI: 0.180, 0.321). In terms of the unstandardized solution, a + 1 difference in baseline adiposity predicted a + 5.3% (IC95%: 1.8%, 9.0%) increase in follow-up HOMA for men and +6.0% IC95%: 4.2%, 7.8% for women. On the other hand, baseline ln(HOMA) was not related to future adiposity in men (−0.060, 95% CI: −0.125, 0.005) but predicted a slight decrease in future adiposity in women (−0.059, 95% CI: −0.097, −0.020) (Figure 1). The latter corresponds to a + 1% difference in baseline HOMA predicting approximately a 0.0025 decrease in adiposity (Table S1, obtained as 0.246/100).

### 3.3. Mediating Role of Adiposity in the Relationship Between Lifestyle Factors and IR

Table 2 shows the estimated direct and indirect effects, including total and specific pathways from our model, between lifestyle exogenous variables and our two outcomes at follow-up in men. Only results for exogenous variables with statistically significant effects are shown. Compared to never-smokers, ex-smoker men had 16.8% (IC95%: 1.6%, 34.2%) higher HOMA GM through all the indirect paths added up together (total indirect effect). A similar effect size was found for current smoking men, with borderline statistical significance (*p* = 0.056). We also found an indirect protective effect between ST and follow-up HOMA through baseline adiposity (−1.2%; IC95%: −2.4%, −0.01%) and a protective effect of PA on HOMA that persisted from baseline to follow-up (specific indirect effect = −10.1% IC95%: −18.5%, −0.8%; total effect = −28.2% IC95%: −41.7%, −11.5%) in men. No significant mediation effects were observed between lifestyle exogenous variables and our outcomes in women.

Model fit indices were CFI = 0.981, TLI = 0.963, RMSEA = 0.053 (IC 90%: 0.035, 0.069), and SRMR = 0.040 for men and CFI = 0.975, TLI = 0.950, RMSEA = 0.058 (IC90%: 0.049, 0.067), and SRMR = 0.042 for women, which indicated that our model was supported by the data and showed acceptable fit based on the RMSEA and very good fit based on the other indexes.

## 4. Discussion

Given the complexity of the relationship between adiposity and HOMA, this study examined their bidirectional relationship. We found an IR prevalence of 23.5% and 55.8% of overweight/obesity, indicating a significant concern for the metabolic health of this population. Although adiposity is traditionally considered a key risk factor for IR, emerging evidence suggests that IR may also contribute to increased adiposity over time. This reciprocal relationship may create a metabolic cycle that disrupts insulin signaling and impairs both innate and adaptive immune responses associated with obesity [22,23,49].

In this study, we found a relationship between baseline adiposity and future HOMA in both sexes, consistent with the findings reported by Xu, C. et al. [50], who reported that abdominal obesity predicts increased IR. However, their results were not stratified by sex. Our study complements these findings by suggesting that sex differences in the expression of central adiposity, particularly in men, may contribute to higher levels of IR compared to women. Notably, we found that baseline HOMA was not related to future adiposity in men. In contrast, among women, higher baseline HOMA-IR was associated with a slight decrease in future adiposity. This finding diverges from Xu, C. et al. [50], who reported a positive association between baseline insulin and follow-up obesity measures without considering sex specific effects. These unexpected sex differences underscore the need for further research to elucidate the underlying mechanisms.

Sex differences in obesity and RI are mediated by complex hormonal and molecular mechanisms that affect the distribution and function of adipose tissue [51]. Estrogen, particularly elevated in premenopausal women, plays a key role in regulating glucose homeostasis. Studies have shown that 17β-estradiol (E2) enhances insulin sensitivity and suppresses hepatic gluconeogenesis via activation of estrogen receptor α (ERα) and downstream signaling through the phosphoinositide 3-kinase–Akt–Foxo1 pathway. This mechanism, identified in animal models, may partially explain the lower incidence of type 2 diabetes in premenopausal women compared with age-matched men, as well as the dysregulation of glucose metabolism observed after menopause due to declining E2 levels [52]. Beyond hepatic effects, estrogens act through their nuclear receptors ERα and ERβ in adipocytes to modulate adipogenesis, inflammation, and mitochondrial function. In animal studies, deletion of ERα leads to increased fat accumulation, elevated levels of circulating and tissue inflammatory markers, impaired glucose tolerance, and IR [53]. Estrogens also regulate the expression of adiponectin, an anti-inflammatory and insulin-sensitizing adipokine, contributing to the higher circulating levels of adiponectin typically found in women [54,55]. Additionally, female mice show a more robust response to adiponectin treatment than males, which has been linked to lower adiponectin receptor expression in male skeletal muscle, potentially regulated by specific proteins and microRNAs [56]. Moreover, sex-specific fat distribution patterns further contribute to metabolic differences. Women tend to accumulate more subcutaneous fat, while men accumulate more visceral adipose tissue (VAT). This difference is not only hormonally mediated but also influenced by genetic factors, such as the expression of genes on the X chromosome that regulate adipose tissue expandability and inflammatory responses [57]. Females predominantly accumulate subcutaneous fat, whereas males accumulate significantly more visceral fat [58]. Visceral fat is metabolically active, characterized by a higher number of large adipocytes, and is associated with greater infiltration of pro-inflammatory macrophages, secretion of cytokines such as TNF-α and IL-6. These features are more pronounced in men and are strongly associated with IR. Adiponectin plays a central protective role [59,60,61]. Its circulating levels are consistently higher in women and contribute to enhanced insulin sensitivity by activating AMP-activated protein kinase (AMPK) and reducing oxidative stress in peripheral tissues [55,62,63]. Importantly, the HOMA-adiposity relationship is complex and dynamic, requiring studies specifically designed to capture changes and more comprehensive IR-adiposity exposure histories. In addition, new indices may be necessary to clarify the mechanisms underlying gender differences [63]. These mechanisms, although supported by existing literature, remain speculative and should be considered as hypotheses that require further investigation and validation in future studies.

Additionally, in this study, we evaluated the relationship between lifestyle factors and adiposity-mediated HOMA. Although several epidemiological studies have explored individual associations between lifestyle factors and adiposity-mediated IR [15,16,17,64], focusing on a single lifestyle factor may be insufficient to fully explain the risk of RI. Analyzing multiple lifestyle factors together could help identify subgroups within the population that are at a higher risk of IR. The results of studies evaluating PA have been contradictory [15,64]. However, in our results, the baseline adiposity acted as a mediator for PA, and PA was strongly related to future HOMA in men. In contrast, our results regarding diet did not support previous findings that examined the relationship between dietary patterns and IR, suggesting that changes in body composition may influence this association [16,17]. These studies highlight the importance of incorporating fruits, vegetables, whole grains, polyunsaturated and monounsaturated fats into healthy eating habits, which are inversely related to IR progression [16,17]. Several studies have reported that individuals with overweight/obesity tend to underreport their energy intake and dietary characteristics, including the consumption of polyunsaturated and monounsaturated fats [65,66], which could bias the association toward the null. In our sample, 40.6% of participants were classified as overweight and 15.2% as obese, which may have contributed to underreporting and limited variability in dietary data. Furthermore, the lack of significant association in our study may be partly explained by the low variability and overall poor quality of diet in our population, reflected by uniformly low consumption of anti-inflammatory foods. According to ENSANUT 2020–2022, the Mexican population reports a low intake of foods recommended for chronic disease prevention [67]. Differences in population characteristics (prevalence of overweight/obesity, diet type, ethnicity) and evaluation methods (energy intake assessment) may also contribute to discrepancies between studies [66,68].

For the other lifestyle factors, we found a relationship between TC and ST with IR. To the best of our knowledge, studies evaluating the relationship between smoking, sleep habits, and HOMA, mediated by adiposity, are scarce; we identified two studies that evaluate these lifestyle factors (smoking and sleep quality) and type 2 diabetes [69,70]. Our results are particularly relevant considering Mexico’s high prevalence of smoking and lack of compliance with PA and sleep recommendations [71,72]. Moreover, a higher percentage of men do not meet the recommendations, which could lead to alterations in physiological pathways, such as circadian misalignment or inflammation, that may result in glucose intolerance and IR [11,12,69,70]. However, it is important to note that some of the effect sizes, such as the borderline significant total indirect effect observed between TC and ln(HOMA) (*p* = 0.051), should be interpreted cautiously. These results are exploratory and require further investigation due to their borderline significance. Regarding the measurement of adiposity, our model suggests that WC was the most reliable indicator in men, whereas BMI was the most reliable indicator in women. In both sexes, body fat proportion was the indicator with the lowest reliability. These results are consistent with a previous ROC analysis study that showed the classification performance of these three indicators for multiple metabolic conditions and metabolic syndrome [44]. Given these results, health services could benefit not only by designing sex-specific treatments, programs, and interventions aimed at reducing adiposity and the risk of developing IR, but also by using this information to help prevent the onset of related metabolic disorders such as hypertension, dyslipidemia, hyperuricemia, cardiovascular disease, T2D, and metabolic syndrome [1,5].

Our study has some strengths and limitations. The longitudinal nature of our data and the measurement of HOMA-IR at two time points allow for a more certain inference on the relationship compared to a cross-sectional one. However, the relatively long 6-year interval between measurements may have missed important intermediate changes in IR and related metabolic factors. This limitation should be considered when interpreting the temporal sequence of effects, as shorter or more frequent assessments might capture dynamic fluctuations more precisely and help clarify causal pathways. Additionally, we observed a high prevalence of overweight/obesity in our population, and this prevalence is expected to be even higher in recent times. In Mexico, ENSANUT has reported that over the last 16 years, the population distribution of BMI and WC has shifted to the right, with a total increase of 21.4% and 6.0%, respectively [7]. We also acknowledge that some participants may have been susceptible to reporting bias, which could favor socially acceptable lifestyles, resulting in estimated parameters with lower magnitudes [68]. Finally, our study population consisted primarily of health workers and their families, which may limit the generalizability of our findings. Nonetheless, this cohort likely approximates urban populations in central Mexico, where lifestyle and metabolic profiles are comparable. We also recognize the potential for residual confounding in our analysis. Although we controlled for several known confounders, unmeasured or residual confounding may still influence the results. Regarding the absence of mediation in women, although we formally tested moderated mediation to explore potential sex differences in the indirect effects, we found no significant differences between men and women. This may be due to insufficient statistical power to detect more subtle differences. Future studies with larger sample sizes and greater statistical power are needed to formally test sex-based differences in mediation processes.

Finally, our analysis included a measurement system combining three indicators of adiposity (BMI, WC, and BFP), which takes into account measurement error and provides higher reliability compared to using just one adiposity indicator at a time. This allows a synthetic presentation of results while improving precision and reducing the number of statistical tests. Structural equation models are attractive, and causal language is generally used for interpretation. In this regard, we employed the term “effect” to describe some of our results as is usually done in the literature; however, it is worth noting that this interpretation requires the assumption that our model is correct (no omitted confounders and correctly specified directionality); there could be other models that may fit the data well or even better [73].

## 5. Conclusions

Our results highlight the importance of considering sex differences in mediation processes, as we observed mediation only in men. This suggests that the factors mediating the relationship between lifestyle factors and adiposity-mediated insulin resistance may operate differently by sex. As far as we know, our study is the first to explore the reciprocal relationship between HOMA-IR and adiposity in Mexican adults. While our findings suggest potential avenues for public health intervention, it is important to note that further research is needed to confirm these associations. Our results support the need for public policies that promote prevention strategies and metabolic health interventions. Given the mediating role of adiposity, comprehensive interventions are warranted to address multiple modifiable lifestyle factors, including increasing physical activity levels, improving sleep quality, and enhancing metabolic health. Such efforts are essential to improve the overall well-being of this population.

## Figures and Tables

**Table 1:** Baseline characteristics of participants from the Health Workers Cohort Study, 2004–2018.

Variable	Total *n* = 1134	Male *n* = 320 (28.0%)	Female *n* = 814 (72.0%)
Age, years ^1^	42.9 ± 11.6	42.4 ± 11.1	43.1 ± 11.8
Education level			
Elemental ^2^	253 (22.3)	57 (17.8)	196 (24.1)
Middle school ^2^	249 (22.0)	80 (25.0)	169 (20.8)
High school or higher ^2^	610 (53.8)	178 (55.6)	432 (53.1)
Missing ^2^	22 (1.9)	5 (1.6)	17 (2.0)
Glucose, mmol/L ^3^	4.9 [4.6, 5.3]	5.2 [4.9, 5.6]	4.9 [4.6, 5.2]
Insulin, UI/L ^3^	7.5 [3.2, 13.3]	9.2 [4.0, 17.5]	6.6 [2.8, 12.1]
HOMA ^3^	1.6 [0.7, 3.1]	2.1 [0.9, 4.3]	1.4 [0.6, 2.7]
IR-HOMA, ≥3.2	266 (23.5)	115 (35.9)	151 (18.6)
BMI ^1^, kg/m^2^	26.0 ± 4.0	26.5 ± 3.7	25.8 ± 4.1
Overweight ^2^	460 (40.6)	156 (48.8)	304 (37.3)
Obesity ^2^	172 (15.2)	56 (15.5)	116 (14.3)
WC ^1^, cm	89.2 ± 11.4	93.6 ± 9.0	87.5 ± 11.8
Central obesity ^2^	412 (36.3)	57 (17.8)	355 (43.6)
BFP ^1^	39.6 [32.7, 44.7]	30.6 [27.3, 34.5]	42.1 [37.8, 46.3]
Excess ^2^	925 (81.6)	260 (81.3)	665 (81.7)
Physical activity ^1^, min/day	24.9 ± 31.7	31.8 ± 36.3	22.2 ± 29.3
Active ^2^, ≥30 min/day	380 (33.5)	132 (41.2)	248 (30.5)
Tobacco consumption			
Ex-smoker ^2^	291 (25.7)	118 (36.9)	173 (21.3)
Current smoker ^2^	214 (18.9)	82 (25.6)	132 (16.2)
Sleep duration ^3^ h/day	7.3 [6.6, 8.0]	7.1 [6.6, 8.6]	7.3 [6.6, 8.0]
Nap duration, min/day	7.5 [0.0, 32.1]	7.5 [0.0, 34.3]	7.5 [0.0, 32.1]
Yes ^2^	766 (67.5)	234 (73.1)	532 (65.4)
Depression score ^3^, CES-D	9 [4, 16]	8 [3, 14]	10 [5, 18]
Depressive symptoms ^2^, ≥16 points	308 (27.2)	61 (19.1)	247 (30.3)
Dietary inflammatory index (DII) ^3^	−0.78 [−1.50, 0.31]	−0.72 [−1.35, 0.53]	−0.79 [−1.54, 0.21]

^1^ Mean ± SD, ^2^ *n* (%), ^3^ Median [P25, P75]. HOMA. Homeostasis model assessment. BMI. Body mass index. BMI ≥ 25 kg/m^2^. WC. Waist circumference. Central obesity ≥ 102 cm in men and ≥88 cm in women. BFP. Body fat proportion. Excess BFP > 25 in men and >35 in women.

**Table 2:** Direct, specific indirect, total indirect, and total effects between lifestyle variables and adiposity and ln(HOMA) final outcomes.

	Adiposity_2_		ln(HOMA_2_)	
	Estimate [SE]	*p*	Estimate [SE]	*p*
Ex-smoker (ExS)				
Specific indirect effects				
ExS_1_ → ln(HOMA_1_) → Outcome_2_	−0.058 [0.042]	0.170	0.116 [0.057]	0.043
ExS_1_ → Adiposity_1_ → Outcome_2_	0.765 [0.445]	0.085	0.039 [0.027]	0.137
Total indirect	0.707 [0.431]	0.101	0.155 [0.071]	0.029
Direct path	0.134 [0.228]	0.557	−0.043 [0.106]	0.684
Total (Direct + Total Indirect)	0.841 [0.477]	0.078	−0.041 [0.102]	0.685
Current smoker (CS)				
Specific indirect effects				
CS_1_ → ln(HOMA_1_) → Outcome_2_	−0.048 [0.040]	0.240	0.094 [0.062]	0.131
CS_1_ → Adiposity_1_ → Outcome_2_	1.089 [0.491]	0.026	0.056 [0.031]	0.074
Total indirect	1.042 [0.475]	0.028	0.150 [0.079]	0.056
Direct path	0.024 [0.253]	0.925	−0.131 [0.113]	0.246
Total (Direct + Total Indirect)	1.066 [0.528]	0.044	0.019 [0.136]	0.888
Physical activity (PA)				
Specific indirect effects				
PA_1_ → ln(HOMA_1_) → Outcome_2_	0.053 [0.038]	0.161	−0.106 [0.050]	0.034
PA_1_ → Adiposity_1_ → Outcome_2_	−0.465 [0.384]	0.225	−0.024 [0.021]	0.260
Total indirect	−0.412 [0.372]	0.268	−0.130 [0.062]	0.035
Direct path	−0.150 [0.198]	0.450	−0.201 [0.089]	0.023
Total (Direct + Total Indirect)	−0.562 [0.413]	0.174	−0.331 [0.107]	0.002
Sleep time (ST)				
Specific indirect effects				
ST_1_ → ln(HOMA_1_) → Outcome_2_	0.004 [0.006]	0.526	−0.008 [0.011]	0.500
ST_1_ → Adiposity_1_ → Outcome_2_	−0.235 [0.091]	0.010	−0.012 [0.006]	0.048
Total indirect	−0.231 [0.088]	0.009	−0.020 [0.015]	0.179
Direct path	−0.023 [0.047]	0.623	−0.005 [0.021]	0.797
Total (Direct + Total Indirect)	−0.254 [0.097]	0.009	−0.025 [0.025]	0.316

*n* = 320. Structural equation model. PA (≥30 min/day), ST (hours/day). Fit statistics: RMSEA = 0.053 (IC 90%: 0.035, 0.069); *p* (RMSEA < 0.05) = 0.377; SRMR = 0.040; CFI = 0.981; TLI = 0.963.

## Data Availability

The datasets used and analyzed during in this study are available at this link: https://drive.google.com/drive/folders/1jXdj0WSrV7HcWrJw7j3Ah1Uh7gr-u1EX?usp=drive_link (accessed on 28 July 2025).

## Supplementary Materials

The following supporting information can be downloaded at: https://www.mdpi.com/article/10.3390/nu17152497/s1, Figure S1: Flow chart of the Health Workers Cohort Study participants; Table S1: Model results of lifestyle factors on the relationship between adiposity and IR.

## Abbreviations

The following abbreviations are used in this manuscript:

IR	Insulin Resistance
HOMA	Homeostasis model assessment
BMI	Body mass index
WC	Waist circumference
BFP	Body fat proportion
T2D	Type 2 diabetes
NHANES	National Health and Nutrition Examination Survey
SEM	Structural equation modeling
AHEI	Alternate healthy eating index
ENSANUT	National Health and Nutrition Survey
HWCS	Health Workers Cohort Study
IMSS	Mexican Social Security Institute
PA	Physical activity
ST	Sleep time
CES-D	Center for Epidemiological Studies Depression Scale
DS	Depressive symptoms
FFQ	Food frequency questionnaire
SNUT	Nutritional Habits and Nutrient Consumption Assessment System
DII	Dietary inflammatory index
DXA	Dual-energy X-ray absorptiometry
CFI	Comparative Fit Index
TLI	Tucker-Lewis Index
RMSEA	Root Mean Square Error of Approximation
90%CI	90% confidence interval
R^2^	Coefficient of determination
SD	Standard deviation
IQR	Interquartile range
