# Supplementary material for: Cross-Lagged Relationship Between Adiposity and HOMA and Mediating Role of Adiposity Between Lifestyle Factors and HOMA Among in Mexican Health Workers
Source: Nutrients. 2025 Jul 30;17(15):2497. doi: 10.3390/nu17152497 (PMC12348819; doi:10.3390/nu17152497)
Supplement: Supplementary file 1 [file nutrients-17-02497-s001.zip › nutrients-3741766-supplementary.pdf]

Supplementary Materials: Figure S1. Flow chart of the Health Workers Cohort Study participants. Table S1. Model results of lifestyle factors on the relationship between adiposity and IR.

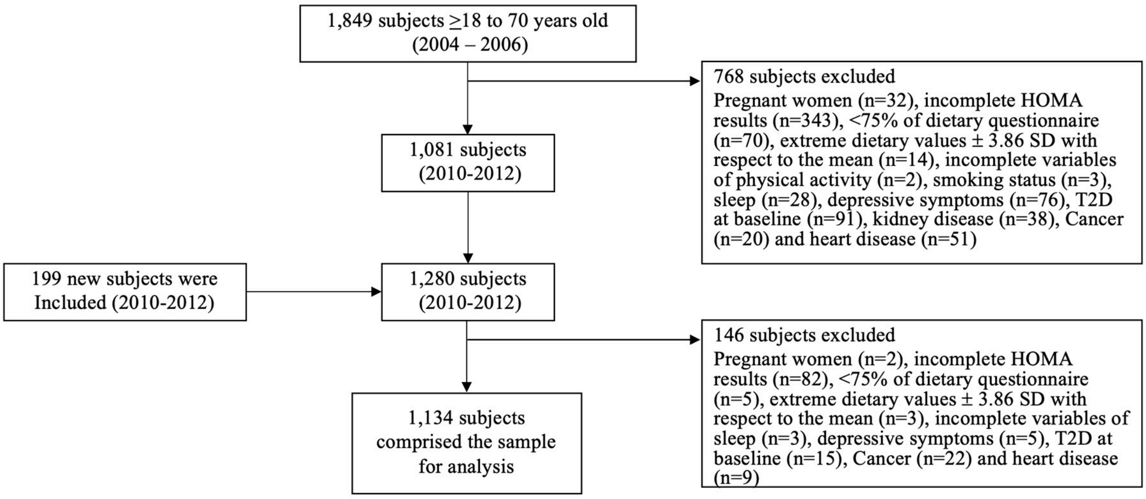

Figure S1. Flow chart of the Health Workers Cohort Study participants.

Table S1. Model results of lifestyle factors on the relationship between adiposity and IR

	**Men**		**Women**	
	**Unstandardized**	**STDY standardization**	**Unstandardized**	**STDY standardization**
	Coef. [SE]	Coef. [SE]	Coef. [SE]	Coef. [SE]
Adiposity$_1$ BY				
BMI$_1$	1	0.890*** [0.017]	1	0.965*** [0.010]
WC$_1$	2.538*** [0.096]	0.940*** [0.016]	2.184*** [0.068]	0.751*** [0.017]
BFP$_1$	1.193*** [0.077]	0.524*** [0.034]	1.169*** [0.039]	0.753*** [0.017]
Adiposity$_2$ BY				
BMI$_2$	1	0.922*** [0.015]	1	0.965*** [0.011]
WC$_2$	2.538*** [0.096]	0.938*** [0.014]	2.184*** [0.068]	0.815*** [0.014]
BFP$_2$	1.193*** [0.077]	0.707*** [0.030]	1.169*** [0.039]	0.759*** [0.015]
Adiposity$_2$ ON				
Adiposity$_1$	1.010*** [0.038]	0.922*** [0.025]	0.996*** [0.024]	0.934*** [0.015]
Adiposity$_2$ ON				
lnHOMA$_1$	-0.204 [0.112]	-0.060 [0.033]	-0.246** [0.082]	-0.059** [0.020]
Age$_1$	-0.623*** [0.102]	-0.172*** [0.028]	-0.675*** [0.085]	-0.158*** [0.020]
DS$_1$	-0.113 [0.250]	-0.031 [0.069]	0.446** [0.163]	0.104** [0.038]
PA$_1$	-0.150 [0.198]	-0.041 [0.055]	-0.316 [0.162]	-0.074 [0.038]
ExS$_1$	0.134 [0.228]	0.037 [0.063]	0.086 [0.187]	0.020 [0.044]
CS$_1$	0.024 [0.253]	0.007 [0.070]	0.097 [0.209]	0.023 [0.049]
ST$_1$	-0.023 [0.047]	-0.006 [0.013]	0.005 [0.064]	0.001 [0.015]
N$_1$	0.160 [0.220]	0.044 [0.061]	-0.059 [0.158]	-0.014 [0.037]
DII$_1$	-0.091 [0.066]	-0.025 [0.018]	0.006 [0.047]	0.001 [0.011]
Adiposity$_1$ ON				
Age$_1$	0.331 [0.195]	0.100 [0.059]	1.280*** [0.145]	0.320*** [0.034]
DS$_1$	-0.500 [0.480]	-0.151 [0.145]	0.316 [0.302]	0.079 [0.075]

PA_1	-0.460 [0.379]	-0.139 [0.114]	-0.296 [0.300]	-0.074 [0.075]
ExS_1	0.757 [0.439]	0.229 [0.132]	0.517 [0.346]	0.129 [0.086]
CS_1	1.078* [0.484]	0.326* [0.144]	0.292 [0.387]	0.073 [0.096]
ST_1	-0.232** [0.089]	-0.070** [0.027]	-0.016 [0.118]	-0.004 [0.030]
N_1	0.633 [0.422]	0.191 [0.127]	0.446 [0.293]	0.111 [0.073]
DII_1	-0.005 [0.128]	-0.002 [0.039]	0.109 [0.087]	0.027 [0.022]
$lnHOMA_2$				
$Adiposity_1$	0.052** [0.017]	0.179** [0.059]	0.058*** [0.009]	0.250*** [0.036]
$lnHOMA_2$				
$lnHOMA_1$	0.405*** [0.050]	0.450*** [0.053]	0.318*** [0.031]	0.349*** [0.032]
Age_1	-0.034 [0.045]	-0.035 [0.047]	-0.035 [0.031]	-0.038 [0.033]
DS_1	-0.032 [0.112]	-0.033 [0.116]	0.009 [0.062]	0.010 [0.066]
PA_1	-0.201* [0.089]	-0.210* [0.092]	-0.026 [0.061]	-0.028 [0.066]
ExS_1	-0.041 [0.102]	-0.043 [0.106]	0.043 [0.071]	0.046 [0.076]
CS_1	-0.131 [0.113]	-0.137 [0.118]	-0.006 [0.079]	-0.007 [0.085]
ST_1	-0.005 [0.021]	-0.006 [0.022]	0.045 [0.024]	0.049 [0.026]
N_1	0.052 [0.099]	0.054 [0.103]	-0.038 [0.060]	-0.042 [0.064]
DII_1	0.042 [0.030]	0.044 [0.031]	0.018 [0.018]	0.020 [0.019]
$lnHOMA_1$				
Age_1	0.003 [0.060]	0.003 [0.056]	0.053 [0.037]	0.052 [0.036]
DS_1	-0.189 [0.150]	-0.177 [0.140]	0.021 [0.078]	0.020 [0.076]
PA_1	-0.262* [0.119]	-0.245* [0.110]	-0.146 [0.077]	-0.143 [0.076]
ExS_1	0.285* [0.136]	0.267* [0.127]	0.084 [0.089]	0.082 [0.088]
CS_1	0.233 [0.151]	0.218 [0.141]	0.165 [0.100]	0.163 [0.098]
ST_1	-0.019 [0.028]	-0.018 [0.026]	-0.009 [0.030]	-0.008 [0.030]
N_1	0.252 [0.132]	0.236 [0.123]	-0.029 [0.075]	-0.029 [0.074]
DII_1	-0.021 [0.040]	-0.020 [0.037]	0.032 [0.023]	0.031 [0.022]
**Covariances**				
$Adiposity_2$ WITH				
$lnHOMA_2$	0.445*** [0.079]	0.353*** [0.052]	0.518*** [0.063]	0.322*** [0.033]
$Adiposity_1$ WITH				
$lnHOMA_1$	1.797*** [0.224]	0.539*** [0.042]	1.615*** [0.152]	0.421*** [0.030]
BMI_1 WITH				
BMI_2	2.397*** [0.354]	0.928*** [0.029]	1.052** [0.309]	0.832*** [0.069]
WC_1 WITH				
WC_2	4.452* [2.030]	0.429*** [0.114]	23.23*** [2.633]	0.455*** [0.035]
BFP_1 WITH				
BFP_2	13.92*** [1.977]	0.502*** [0.048]	11.17*** [0.858]	0.639*** [0.024]
**Intercepts**				
BMI_1	0	0	0	0
WC_1	26.23*** [2.564]	2.94*** [0.325]	31.29*** [1.783]	2.69*** [0.187]
BFP_1	-0.662 [2.091]	-0.088 [0.277]	11.72*** [1.008]	1.887*** [0.187]
BMI_2	0	0	0	0
WC_2	27.85*** [2.625]	2.84*** [0.308]	32.37*** [1.832]	2.83*** [0.200]
BFP_2	-0.133 [2.118]	-0.022 [0.346]	13.09*** [1.041]	1.99*** [0.188]
$lnHOMA_1$	0.415** [0.148]	0.389** [0.141]	0.278*** [0.077]	0.274*** [0.075]
$lnHOMA_2$	-0.788 [0.446]	-0.820 [0.460]	-0.933*** [0.222]	-1.008*** [0.234]
$Adiposity_1$	25.81*** [0.480]	7.80*** [0.422]	25.36*** [0.298]	6.33*** [0.196]
$Adiposity_2$	0.399 [0.994]	0.110 [0.277]	0.978 [0.629]	0.229 [0.151]

**Residual Variances**				
BMI$_1$	2.873 [0.401]	0.208 [0.030]	1.190 [0.340]	0.069 [0.020]
WC$_1$	9.305 [2.362]	0.117 [0.031]	59.165 [3.567]	0.436 [0.025]
BFP$_1$	41.074 [3.450]	0.725 [0.035]	16.694 [0.996]	0.433 [0.026]
BMI$_2$	2.324 [0.400]	0.150 [0.028]	1.346 [0.393]	0.069 [0.021]
WC$_2$	11.571 [2.448]	0.120 [0.026]	44.070 [2.923]	0.337 [0.023]
BFP$_2$	18.690 [1.688]	0.500 [0.042]	18.290 [1.054]	0.424 [0.023]
lnHOMA$_1$	1.088 [0.086]	0.955 [0.023]	1.021 [0.051]	0.987 [0.008]
lnHOMA$_2$	0.596 [0.047]	0.647 [0.043]	0.635 [0.032]	0.741 [0.027]
Adiposity$_1$	10.209 [1.000]	0.933 [0.028]	14.390 [0.836]	0.897 [0.021]
Adiposity$_2$	2.664 [0.264]	0.203 [0.023]	4.084 [0.285]	0.224 [0.017]
**R squared**				
Adiposity$_1$	0.067 [0.028]		0.103 [0.021]	
BMI$_1$	0.792 [0.030]		0.931 [0.020]	
WC$_1$	0.883 [0.031]		0.564 [0.025]	
BFP$_1$	0.275 [0.035]		0.567 [0.026]	
Adiposity$_2$	0.797 [0.023]		0.776 [0.017]	
BMI$_2$	0.850 [0.028]		0.931 [0.021]	
WC$_2$	0.880 [0.026]		0.663 [0.023]	
BFP$_2$	0.500 [0.042]		0.576 [0.023]	
lnHOMA$_1$	0.045 [0.023]		0.013 [0.008]	
lnHOMA$_2$	0.353 [0.043]		0.259 [0.027]	
**Model Fit**				
CFI	0.981		0.975	
TLI	0.963		0.950	
RMSEA (90% CI)	0.053 (0.035, 0.069)		0.058 (0.049, 0.067)	
SRMR	0.040		0.042	
**Sample size**	320		814	

*p<0.05, **p<0.01, ***p<0.001 (not shown for residual variances and R squared)

BMI: body mass index (kg/m^2); WC: waist circumference (cm); BFP: total body fat mass proportion; DS: depressive symptoms (≥ 16 points); PA: physical activity (≥ 30 min/day); ExS: Ex-smoker; CS: current smoker; ST: sleep time (hours/day) centered at its mean; N: nap (yes); DII: dietary inflammatory index. Age was standardized.

CFI: Comparative Fit Index; TLI: Tuker-Lewis Index; RMSEA: Root Mean Square Error of Approximation; SRMR: Standardized Root Mean Square Residual
